# High-Output-Current Boron-Doped Single-Crystal Diamond MOSFETs with a Thin Boron-Doped Epitaxial Layer

**DOI:** 10.3390/nano16150915

**Published:** 2026-07-25

**Authors:** Jiali Wang, Ruozheng Wang, Liangshun Qu, Genqiang Chen, Feng Wen, Hongxing Wang

**Affiliations:** 1 Key Laboratory of Physical Electronics and Devices, Ministry of Education, Xi’an Jiaotong University, Xi’an 710049, China; 2 School of Electronic Science and Engineering, Xi’an Jiaotong University, Xi’an 710049, China; 3 State Key Laboratory for Mechanical Behavor of Materials, Xi’an Jiaotong University, Xi’an 710049, China

**Keywords:** boron-doped diamond, MOSFETs, thin epitaxial layer, high-output current, temperature-dependent characteristics

## Abstract

High-output-current boron-doped diamond (B-diamond) metal–oxide–semiconductor field-effect transistors (MOSFETs) with a modulated boron-doped epitaxial layer were fabricated. An intrinsic diamond epitaxial layer was deposited on the single-crystal diamond substrate as a buffer layer, and plasma-enhanced chemical vapor deposition (PECVD) SiO_2_ was employed as both the gate dielectric and passivation layer. The boron-doped epitaxial layer has a thickness of approximately 500 nm and a boron concentration in the range of 10^17^–10^18^ cm^−3^. The B-diamond MOSFETs showed clear p-channel operation, with a maximum output current of −0.18 mA/mm at room temperature. When the temperature was increased to 150 °C, the maximum output current increased to −1.05 mA/mm, while the on-resistance decreased from 413.91 to 12.13 kΩ·mm. The on/off ratio remains approximately 10^5^ over the measured temperature range. In addition, the device exhibited a breakdown voltage of −347 V at a gate-to-drain spacing of 12.5 μm, and the simulation results showed that the peak electric field was mainly concentrated near the drain-side gate edge of the passivation layer. These results indicated that, for the B-diamond MOSFETs, a balanced epitaxial layer thickness and boron concentration were essential for achieving sufficient channel conduction as well as effective gate control ability.

## 1. Introduction

Diamond-based electronic devices are highly promising for low-loss and high-frequency applications due to the excellent intrinsic properties of diamond [1,2,3]. The material features an ultra-wide bandgap of 5.47 eV, a critical breakdown field of 10 MV/cm, and an exceptional thermal conductivity reaching 22 W/cm·K. Furthermore, diamond exhibits high carrier mobilities, with an electron mobility of 4500 cm^2^/V·s and a hole mobility of 3800 cm^2^/V·s [4,5]. Undoped diamond is an excellent insulator with an electrical resistivity higher than 10^15^ Ω·cm, although its conductivity can be increased by introducing dopants to form p-type or n-type diamond. Therefore, intentional channel formation is essential for realizing diamond MOSFETs. To leverage these advantages, p-type channel layers based on hydrogen-terminated diamond (H-diamond), silicon-terminated diamond (Si-diamond) [6,7], and boron-doped diamond are employed in the fabrication of high-performance metal–oxide–semiconductor field-effect transistors (MOSFETs) [8,9,10,11].

To date, H-diamond MOSFETs remain the mainstream devices in current research and applications [12,13,14,15]. The drain current, cut-off frequency, and breakdown voltage have reached 1.35 A/mm [16], 70 GHz [17], and 3326 V [18], respectively. However, the thermal stability of such devices still poses significant challenges. In Si-diamond, the C–Si bonds induce hole accumulation, similar to the C–H bonds in H-diamond. Consequently, Si-diamond MOSFETs can operate effectively at 300 and 400 °C, demonstrating an excellent maximum drain current and maximum transconductance of −124 mA/mm and 4100 μS/mm [6], respectively. Nevertheless, operation at such high temperatures still leads to the degradation of electrical properties, including a significant decrease in the on/off ratio. In contrast, B-diamond MOSFETs are primarily suitable for extreme environments [19]. Compared with H-diamond MOSFETs, whose channel conductivity relies on surface transfer doping or surface adsorbates, the conducting channel in B-diamond MOSFETs is formed by substitutional boron acceptors in the epitaxial layer. Therefore, B-diamond MOSFETs are expected to be less sensitive to ambient surface adsorbates and environmental variations, which is beneficial for high-temperature and long-term stable operation.

In recent years, efforts have been devoted to developing diamond MOSFETs with oxygen-terminated surfaces on B-diamond channels [8,9,11,20,21]. However, due to the activation energy of boron dopants (370 meV), which is higher than the thermal energy at room temperature (26 meV) [22], B-diamond MOSFETs operate at low-output currents. The fundamental issue appears to be the low hole density of the devices, which leads to suboptimal current density and electrical conductivity [23]. Previous studies have shown that the drain current can be enhanced by increasing the B-diamond epitaxial layer thickness and carrier concentration. Liu et al. reported that, at a fixed boron concentration (N_boron_), both |I_D,max_| and V_TH_ increased with increasing B-diamond layer thickness (t_boron_). Similarly, for devices with comparable t_boron_, higher N_boron_ resulted in larger |I_D,max_| and V_TH_ [24]. However, an excessively thick or heavily doped B-diamond layer makes the device difficult to turn off. In addition, a thick and highly doped single-crystal diamond layer requires longer epitaxial growth and may introduce additional crystalline defects, which can degrade material quality and affect reliable device operation. Therefore, selecting a suitable B-diamond epitaxial thickness and boron concentration is important for balancing channel conduction and gate controllability.

In this work, a ring-gate device structure was adopted for B-diamond MOSFETs to suppress edge leakage and enhance the output current. The boron-doped epitaxial layer thickness and boron concentration used in this work provided sufficient channel conduction while maintaining effective gate modulation. A PECVD (Trion Technology, Clearwater, FL, USA)-grown SiO_2_ layer was used as both the gate dielectric and passivation layer, providing gate insulation and surface passivation for the B-diamond MOSFETs. Selective etching was then performed to open the electrode contact windows for source/drain formation. The fabricated devices were systematically characterized in terms of electrical, temperature-dependent, and breakdown characteristics, demonstrating their potential for high-temperature diamond electronic devices.

## 2. Experimental

**Figure 1:**
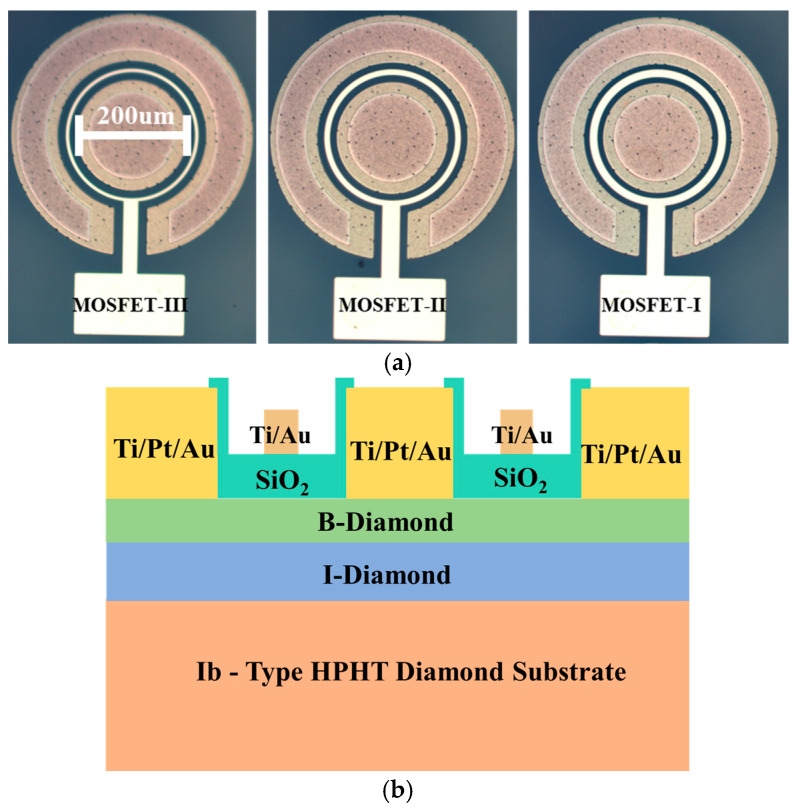
(**a**) Optical microscope images and (**b**) schematic diagram of the B-diamond MOSFET.

Figure 1a,b show an optical microscope image and schematic diagram of the B-diamond MOSFET, respectively. The diameter of the drain electrode is 200 μm. The gate width (W_G_) can be calculated as 745 μm. The devices with gate lengths (L_G_) of 12 μm, 8 μm, and 4 μm are named MOSFET-I, MOSFET-II, and MOSFET-III, respectively. The L_G_, source-to-gate spacings (L_S−G_), and gate-to-drain spacings (L_D−G_) values are 12, 12.5, and 12.5 µm for MOSFET-I, 8, 14.5, and 14.5 µm for MOSFET-II, and 4, 16.5, and 16.5 µm for MOSFET-III, respectively.

**Figure 2:**
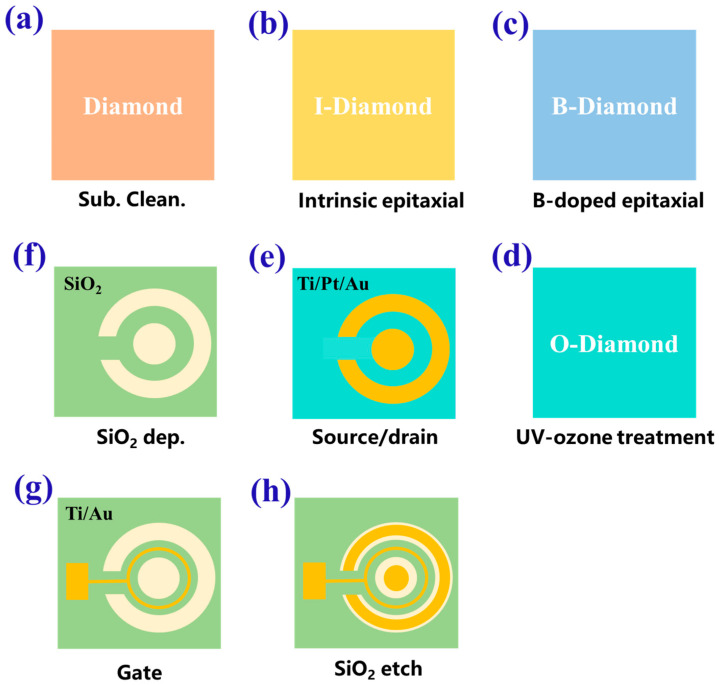
Fabrication process for B-diamond MOSFETs: (**a**) diamond cleaning, (**b**) I-diamond growth, (**c**) B-diamond growth, (**d**) UV-ozone treatment, (**e**) Ti/Pt/Au source/drain electrode formation, (**f**) SiO_2_ deposition for gate oxide, (**g**) Ti/Au gate electrode formation, and (**h**) opening windows for the electrodes.

Figure 2 illustrates the fabrication process for the B-diamond MOSFETs. First, the 3 × 3 × 0.5 mm^3^ Ib-type (100) HPHT single-crystal diamond substrate was boiled in a mixed solution of H_2_SO_4_ and HNO_3_ at 250 °C for 1 h to clean the diamond surface [Figure 2a]. The intrinsic diamond (I-diamond) layer was grown without supplying a boron source, followed by the growth of the B-diamond epitaxial layer in the in situ MPCVD (Cornes Technologies Ltd., Tokyo, Japan) system [Figure 2b,c]. The B-diamond surface was then treated with ultraviolet ozone (UV/O_3_) for 30 min to change the hydrogen-terminated surface to an oxygen-terminated surface [Figure 2d]. The source/drain electrodes, consisting of a Ti/Pt/Au multilayer (50/50/100 nm), were evaporated on the B-diamond by an electron-gun evaporation system (EB) (ULVAC, Kanagawa, Japan) [Figure 2e]. The chamber pressure was approximately 5 × 10^−4^ Pa, and the evaporation rate was 1 Å/s. Then, the source/drain electrodes were annealed at 550 °C for 20 min in an Ar atmosphere using a rapid thermal annealing system to form ohmic contacts. A 200 nm SiO_2_ gate oxide was deposited by PECVD at 350 °C [Figure 2f]. Finally, a Ti/Au (50/100 nm) bilayer was also evaporated by EB as the gate electrode [Figure 2g]. Windows for accessing the electrodes were opened by etching the SiO_2_ film using a capacitively coupled plasma reactive ion etching system (Oxford Instruments, Abingdon, UK) in an atmosphere of CF_4_, Ar, and O_2_ [Figure 2h]. After device fabrication, the electrical characteristics were measured using an Agilent B1505A semiconductor device analyzer (Keysight Technologies Inc., Santa Clara, CA, USA).

## 3. Results

**Figure 3:**
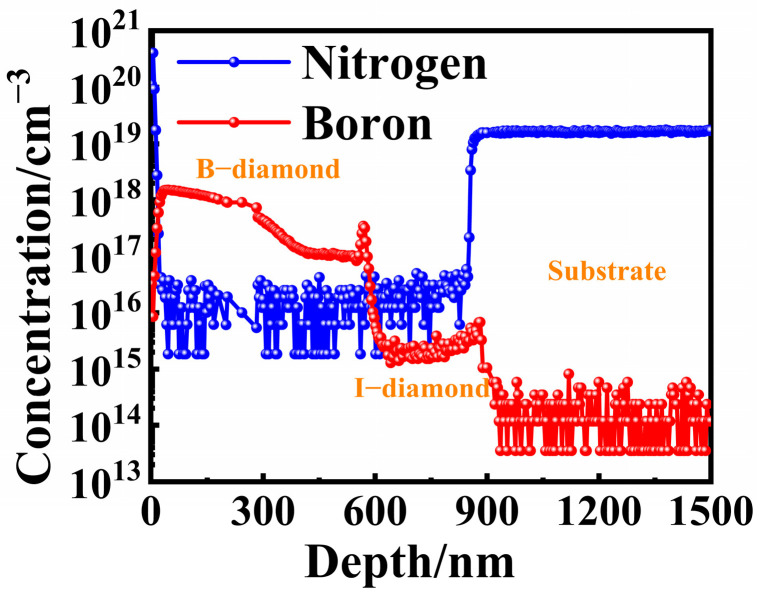
SIMS depth profiles of boron and nitrogen concentrations in the B-diamond epitaxial layer, I-diamond buffer layer, and HPHT diamond substrate.

The depth profiles of boron and nitrogen concentrations were characterized by secondary ion mass spectrometry (SIMS), as shown in Figure 3. The region from 0 to approximately 500 nm corresponds to the B-diamond epitaxial layer, where the boron concentration is in the range of 10^17^–10^18^ cm^−3^. The region from approximately 500 to 900 nm corresponds to the I-diamond buffer layer, where the boron concentration decreases sharply. In the deeper region from approximately 900 to 1500 nm, corresponding to the HPHT diamond substrate, the boron concentration approaches the background level. The nitrogen concentration remains relatively low in the B-diamond and I-diamond epitaxial layers, but increases sharply in the HPHT substrate region. This result is consistent with the native nitrogen impurity commonly present in Ib-type HPHT diamond substrates. Therefore, the I-diamond buffer layer, with relatively low boron and nitrogen concentrations, acts as a nominally intrinsic transition layer between the B-diamond active channel and the HPHT substrate, reducing the influence of nitrogen-related impurities on the upper B-diamond channel.

**Figure 4:**
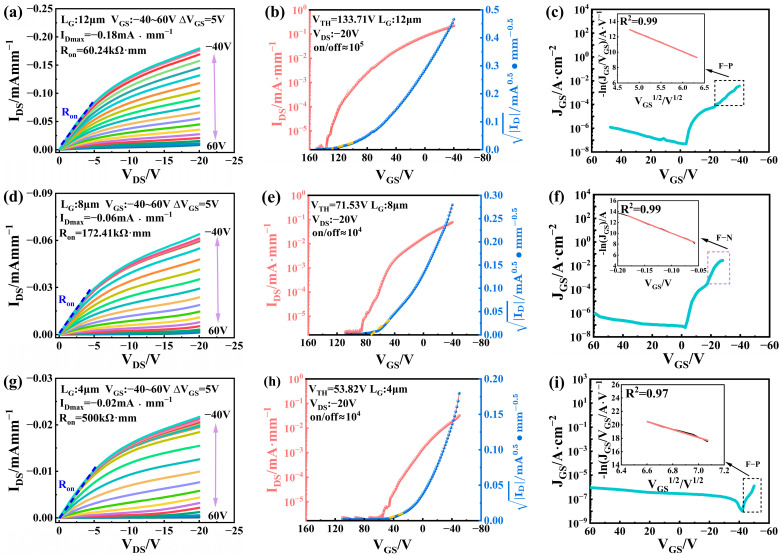
Output, transfer, and gate leakage characteristics of the fabricated B-diamond MOSFETs. (**a**–**c**) MOSFET-I, (**d**–**f**) MOSFET-II, and (**g**–**i**) MOSFET-III. (**a**,**d**,**g**) show the I_D_–V_D_ output characteristics; (**b**,**e**,**h**) show the I_D_–V_GS_ transfer characteristics with √|I_D_| plots for V_TH_ extraction; and (**c**,**f**,**i**) show the gate leakage characteristics with high-field leakage fitting in the insets.

Figure 4a,d,g show the drain current–drain voltage (I_D_–V_D_) characteristics of MOSFET-I, -II, and -III, respectively. The V_GS_ values vary from −40.0 to 60.0 V in steps of 5.0 V. The devices show clear p-channel operation. The maximum drain current values (I_D,max_) for MOSFET-I, -II, and -III are −0.18, −0.06, and −0.02 mA/mm, with corresponding on-resistance (R_on_) values of 60.24, 172.41, and 500 kΩ·mm, respectively. Figure 4b,e,h show the I_D_–V_GS_ characteristics of MOSFET-I, -II, and -III, respectively. The threshold voltage (V_TH_) was extracted from the transfer characteristics measured at V_DS_ = −20 V using the linear extrapolation method. Since the devices are p-channel MOSFETs, the absolute value of the drain current was used for the extraction. The nearly linear region of the √|I_D_|-V_GS_ curve in the strong-conduction regime was fitted, and V_TH_ was obtained from the intercept of the fitted line with the V_GS_-axis. The extracted V_TH_ values for MOSFET-I, -II, and -III are 133.71, 71.53, and 53.82 V, and the corresponding transconductances (g_m_) are 6.21, 2.80, and 2.41 μS·mm^−1^, respectively. The on/off ratios of the three MOSFETs are in the range of 10^4^–10^5^. The variations in |I_D,max_|, R_on_, and V_TH_ are related to the combined effects of gate length and access-region spacing. As L_G_ decreases, L_S−G_ and L_D−G_ increase, leading to larger access resistance, reduced output current, and increased R_on_. Meanwhile, the extracted V_TH_ gradually decreases from MOSFET-I to MOSFET-III.

The relatively high and different V_TH_ values are attributed to the bulk-doped B-diamond channel [25] and device geometry. The B-diamond epitaxial layer serves as the active hole-conducting channel. Therefore, residual holes may remain in the doped layer even under positive gate bias, and a relatively large gate bias is required to deplete or modulate the channel charge. The threshold voltage can be qualitatively described by the depletion approximation:

(1) $$V_{\mathrm{TH}}{=V}_{\mathrm{FB}}+\frac{{\mathrm{qN}}_{A}t_{\mathrm{boron}}^{2}}{\varepsilon_{\mathrm{dia}}\varepsilon_{0}}$$

The drain current in the saturation region can be expressed as:

(2) $$I_{D,\max}=\frac{W_{G}}{{2L}_{G}}\mu_{\mathrm{eff}}C_{\mathrm{ox}}{{(V}_{\mathrm{GS}}{-V}_{\mathrm{TH}})}^{2}$$

where N_A_ is the acceptor concentration, t_boron_ is the B-diamond thickness, V_FB_ is the flat-band voltage, q is the elementary charge, ϵ_dia_ is the relative permittivity of diamond, ϵ_0_ is the vacuum permittivity, and μ_eff_ is the effective mobility for the B-diamond channel.

Equations (1) and (2) indicate that increasing N_A_ or t_boron_ increases the channel charge that must be depleted by the gate, resulting in a higher V_TH_. At the same time, a higher boron concentration or a thicker B-diamond layer can provide more holes for channel conduction, leading to an increased |I_D,max_|. Therefore, |I_D,max_| and V_TH_ tend to increase simultaneously with increasing boron concentration or B-diamond thickness, reflecting the trade-off between current capability and gate controllability.

Figure 4c,f,i show the gate leakage characteristics of the three B-diamond MOSFETs. The gate leakage current density remains relatively low over most of the gate-bias range, indicating that the PECVD SiO_2_ layer provides effective gate insulation. At high negative gate bias, J_GS_ increases noticeably, which is attributed to enhanced high-field leakage through the gate dielectric. The insets show the corresponding high-field fitting results, suggesting that Poole–Frenkel emission and Fowler–Nordheim tunneling may be involved in the leakage process. Therefore, further improvement of the SiO_2_/B-diamond interface quality is still needed to suppress trap-assisted leakage and improve device reliability.

To further verify the reproducibility of the device characteristics, additional B-diamond MOSFETs fabricated on the same sample were also measured, as summarized in Table 1. These devices show clear p-channel operation, although variations in |I_D,max_|, R_on_, V_TH_, on/off ratio, and g_m_ are observed. Such device-to-device variation may be related to local boron doping nonuniformity, contact resistance, access-region spacing, and the PECVD SiO_2_/B-diamond interface quality. Nevertheless, the additional measured devices confirm that the MOSFET characteristics are reproducible on the same sample.

**Figure 5:**
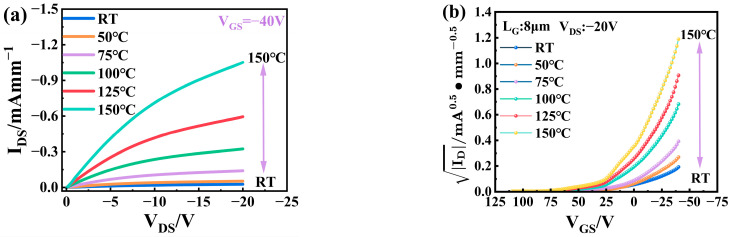
(**a**) I_D_–V_D_ and (**b**) I_D_–V_GS_ characteristics for the B-diamond MOSFETs operating from RT to 150 °C. The V_GS_ in (**a**) is kept at –40.0 V. The V_DS_ in (**b**) is kept at –20.0 V.

**Figure 6:**
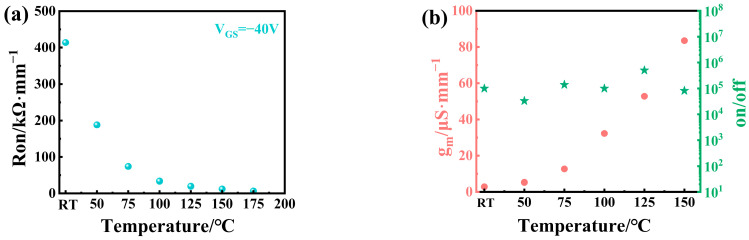
(**a**) R_on_ and (**b**) g_m_ and on/off ratio of the B-diamond MOSFETs as functions of operating temperature.

To further evaluate the high-temperature operation of the B-diamond MOSFETs, the electrical characteristics were measured from RT to 150 °C. Figure 5a shows the I_D_–V_D_ characteristics measured at different temperatures with V_GS_ = −40 V. At an operating temperature of 150 °C, the B-diamond MOSFET continues to operate well. The |I_D,max_| values for the B-diamond operating at RT, 50, 75, 100, 125, and 150 °C are 0.03, 0.05, 0.14, 0.32, 0.60 and 1.05 mA·mm^−1^, respectively, which indicates that |I_D,max_| at 150 °C is approximately 35 times higher than that at RT. Correspondingly, their R_on_ values are determined to be 413.91, 188.11, 74.05, 33.74, 19.82 and 12.13 kΩ mm, respectively, as shown in Figure 6a.

Figure 5b shows the I_D_–V_GS_ characteristics for the B-diamond MOSFETs operating from RT to 150 °C. With increasing temperature, V_TH_ shifts noticeably, indicating changes in the hole concentration and channel charge distribution in the B-diamond layer. Figure 6b summarizes the g_m_ and on/off ratio as functions of temperature. The g_m_ values measured at RT, 50, 75, 100, 125, and 150 °C are 2.87, 5.32, 12.71, 32.30, 52.84 and 83.53 μS·mm^−1^, respectively. Notably, g_m_ increases by more than 29 times from RT to 150 °C. The on/off ratios remain stable at approximately 10^5^, indicating that gate modulation is maintained during the temperature-dependent measurement.

**Figure 7:**
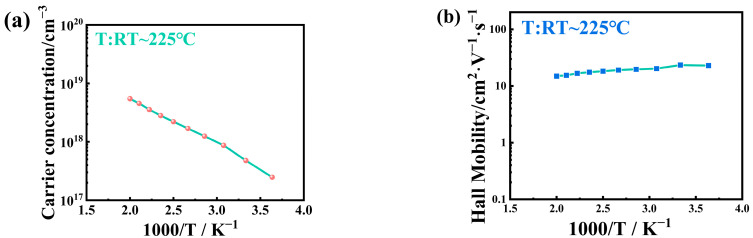
Temperature-dependent Hall measurement results of the B-diamond epitaxial layer from RT to 225 °C: (**a**) carrier concentration and (**b**) Hall mobility.

To clarify the origin of the enhanced current at elevated temperatures, Hall measurements were performed on the B-diamond epitaxial layer from RT to 225 °C. As shown in Figure 7a, the carrier concentration increases continuously with increasing temperature, which can be attributed to the thermal activation of boron acceptors in diamond. In contrast, Figure 7b shows that the Hall mobility decreases only slightly over the same temperature range, mainly due to enhanced phonon scattering at higher temperatures. This trend indicates that the increase in hole concentration plays an important role in the temperature-dependent transport behavior. The channel conductivity can be approximately expressed as σ = qpμ, where q, p, and μ are the elementary charge, hole concentration, and hole mobility, respectively. Therefore, although the mobility slightly decreases, the increase in hole concentration dominates the transport behavior, leading to enhanced channel conductivity and reduced channel resistance. This result explains the continuous increase in |I_D,max_| with increasing temperature. In addition, the temperature-dependent change in carrier concentration may also contribute to the observed shift in V_TH_.

**Figure 8:**
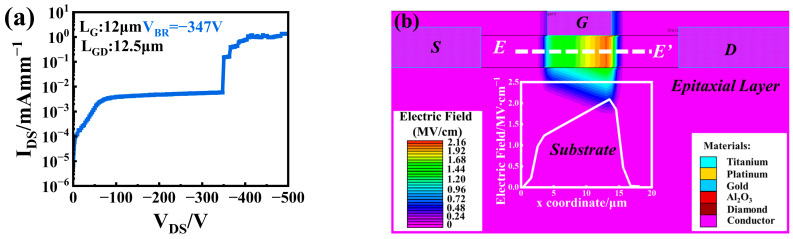
B-diamond MOSFET (**a**) breakdown voltage and (**b**) electric field simulation.

The breakdown voltage (V_BR_) of the B-diamond MOSFET was measured to be −347 V, as shown in Figure 8a. Dividing this value by L_GD_ = 12.5 μm gives an average lateral breakdown field of approximately 0.28 MV/cm. This value is much lower than the intrinsic critical electric field of diamond, indicating that the breakdown is unlikely to be limited by the intrinsic bulk breakdown of diamond.

Figure 8b shows the simulated electric-field distribution inside the device at a breakdown voltage of −347 V. The simulation was performed using the ATLAS module of Silvaco TCAD (v2018). The device structure was constructed according to the fabricated B-diamond MOSFET, including the Ti/Pt/Au source/drain electrodes, gate electrode, SiO_2_ dielectric layer, B-diamond epitaxial layer, intrinsic diamond buffer layer, and diamond substrate. Carrier transport, recombination, mobility, and impact ionization models were included in the simulation. The peak electric field is mainly concentrated near the drain-side gate edge and extends toward the SiO_2_/B-diamond interface, indicating that the breakdown is unlikely to be limited by intrinsic bulk diamond breakdown. The inset displays the electric-field distribution along the EE′ line within the SiO_2_ dielectric layer.

Further improvement of the breakdown voltage requires optimization of the device geometry and field management. Increasing the gate-to-drain spacing can reduce the average lateral electric field and redistribute the field in the access region. Edge termination and field-plate structures have been reported to suppress local electric-field crowding and improve reverse breakdown characteristics in diamond power devices [26,27,28]. In addition, the SiO_2_ thickness should be optimized, since a thicker oxide can reduce the electric field in the gate dielectric and improve dielectric reliability, whereas an excessively thin oxide may increase gate leakage, charge trapping, and premature dielectric breakdown.

To provide a clearer evaluation, Table 2 compares the structural parameters and electrical characteristics of representative B-diamond MOSFETs reported in the literature. The 500 nm B-diamond epitaxial layer used in this work is not claimed to be the thinnest among all reported devices. In a bulk-doped B-diamond MOSFET, the B-diamond layer serves as an active hole-conducting channel; therefore, its thickness must be sufficient for channel conduction while still allowing effective gate control. Compared with thicker B-diamond layers, a thinner active layer can reduce growth time and improve gate controllability, but the near-surface channel may be more sensitive to the SiO_2_/B-diamond interface quality. Therefore, long-term reliability, such as prolonged high-temperature operation and dielectric reliability, still requires further investigation. This comparison also shows that the present device maintains an on/off ratio of approximately 10^5^. The values of |I_D,max_| and g_m_ increase from 0.03 mA/mm and 2.87 μS/mm at RT to 1.05 mA/mm and 83.53 μS/mm at 150 °C, respectively. These results show that the 500 nm B-diamond layer provides sufficient channel conduction and stable gate modulation during temperature-dependent operation.

## 4. Conclusions

Diamond MOSFETs were fabricated on a 500 nm thick boron-doped diamond epitaxial layer with a doping concentration of 10^17^–10^18^cm^−3^. The B-diamond layer was grown on an intrinsic diamond buffer layer by MPCVD, and SiO_2_ was used as both the gate dielectric and passivation layer. The fabricated devices exhibit clear p-type MOSFET characteristics, with an on/off ratio of approximately 10^5^ at room temperature and maximum output currents of −0.18, −0.06, and −0.02 mA/mm for MOSFET-I, MOSFET-II, and MOSFET-III, respectively. At 150 °C, the maximum output current increases to −1.05 mA/mm, while the on/off ratio remains around 10^5^. Hall measurements indicate that the carrier concentration in the B-diamond layer increases with temperature, whereas the mobility decreases only slightly, suggesting that the enhanced drain current mainly originates from thermally activated holes from boron acceptors. A breakdown voltage of −347 V was obtained for the device with L_GD_ = 12.5 μm, and electric-field simulation shows that the peak field is mainly concentrated near the drain-side gate edge. These results show that the 500 nm B-diamond epitaxial layer can provide sufficient channel conduction while maintaining gate modulation. Further optimizations, such as p–n heterojunction, device structure, and field-plate management, are still needed to improve the overall device performance and breakdown reliability.

## Figures and Tables

**Table 1:** Electrical characteristics of additional B-diamond MOSFETs fabricated on the same sample.

L_G_ (μm)	L_SG_ (μm)	L_DG_ (μm)	I_Dmax_ (mA·mm^−1^)	R_on_ (kΩ·mm)	V_TH_ (V)	On/Off	g_m_ (μS·mm^−1^)
12	12.5	12.5	−0.10	91	>110	10^3^	3.78
8	14.5	14.5	−0.02	770	57.5	10^4^	1.57
8	14.5	14.5	−0.03	360	76.28	10^6^	2.87
4	16.5	16.5	−0.08	116	85	10^6^	4.01

**Table 2:** Comparison of structural parameters and device performance of B-diamond MOSFETs.

	t_boron_ (nm)	N_boron_ (cm^−3^)	L_G_ (μm)	Operating Temperature (°C)	|I_D,max_| (mA/mm)	On/Off Ration	V_TH_ (V)	g_m,max_ (μS/mm)	V_BR_ (V)
[8]	230	1.75 × 10^17^	/	RT	0.0019	~10^4^	7.0	/	200
[11]	700	~10^17^	7.0	RT	0.49	/	63.2	18.7	/
[29]	2650	~10^16^	2.6	RT	1.2	>10^9^	63.8	29.0	/
				300 °C	10.9		31.2	215.7	/
[30]	2000	~10^16^	5.6	RT	0.1097	~10^8^	58.8	4.1	/
[19]	825	~4 × 10^15^	4.8	RT	0.0039	1.9 × 10^5^	8.3	0.9	/
				400 °C	0.1774	5.5 × 10^6^	4.1	23.1	/
[31]	150	10^16^~10^17^	3	RT	0.00012	>10^4^	−8.0	/	>1700
[24]	1514	~10^17^	2.8	RT	4.7	/	320.2	/	/
				300 °C	76.9	/	1143.2	/	/
				400 °C	109.2	/	895.1		/
This work	500	10^17^~10^18^	8	RT	0.03	~10^5^	76.28	2.87	347
				150 °C	1.05	~10^5^	83.53	83.53	

## Data Availability

The original contributions presented in this study are included in the article/Supplementary Material. Further inquiries can be directed to the corresponding authors.

## Supplementary Materials

The following supporting information can be downloaded at: https://www.mdpi.com/article/10.3390/nano16150915/s1, Figure S1: CTLM characteristics of Ti/Pt/Au contacts on B-diamond after Ar annealing: (a) I–V curves with different gap spacings and (b) extracted total resistance as a function of gap spacing; Figure S2: CTLM characteristics of Ti/Pt/Au contacts on B-diamond after vacuum annealing: (a) I–V curves with different gap spacings and (b) extracted total resistance as a function of gap spacing; Figure S3: Forward/reverse C–V characteristics and C−2–V fitting of the post-etched B-diamond MOSFET structure; Figure S4: Forward/reverse transfer characteristics of the B-diamond MOSFETs: (a) MOSFET-I; (b) MOSFET-II; and (c)MOSFET-III.
