# Supplementary material for: High-Output-Current Boron-Doped Single-Crystal Diamond MOSFETs with a Thin Boron-Doped Epitaxial Layer
Source: Nanomaterials (Basel). 2026 Jul 25;16(15):915. doi: 10.3390/nano16150915 (PMC13468557; doi:10.3390/nano16150915)
Supplement: Supplementary file 1 [file nanomaterials-16-00915-s001.zip › nanomaterials-4419023-supplementary.pdf]

supporting information

S1: To clarify the contact behavior, we performed CTLM measurements for Ti/Pt/Au contacts after annealing in Ar and vacuum environments, as shown in Figure S1 and Figure S2, respectively.

The CTLM I–V curves with different gap spacings show nearly linear and symmetric behavior, indicating that the Ti/Pt/Au/B-diamond contacts are not strongly rectifying. The total resistance was extracted from the slope of the I–V curves for each gap spacing and then plotted as a function of gap spacing after CTLM geometry correction. From the linear fitting, the contact resistance and transfer length were obtained, and the specific contact resistivity was estimated using

$$\rho_c = R_{sh} L_T^2$$

where R_{sh} is the sheet resistance and L_T is the transfer length.

The extracted specific contact resistivity is 4.31×10^{-3} Ω·cm² for the Ar-annealed contact and 1.06×10^{-2} Ω·cm² for the vacuum-annealed contact. The lower contact resistivity after Ar annealing indicates that the Ar atmosphere improves the metal/B-diamond contact behavior. Therefore, the Ohmic-contact claim is supported by the nearly linear CTLM I–V curves and the reduced contact resistivity after Ar annealing.

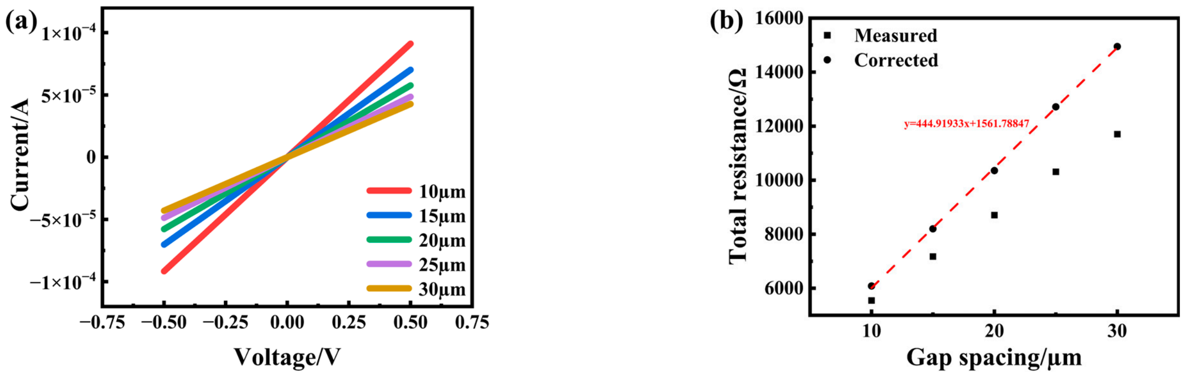

Figure S1. CTLM characteristics of Ti/Pt/Au contacts on B-diamond after Ar annealing: (a) I–V curves with different gap spacings and (b) extracted total resistance as a function of gap spacing.

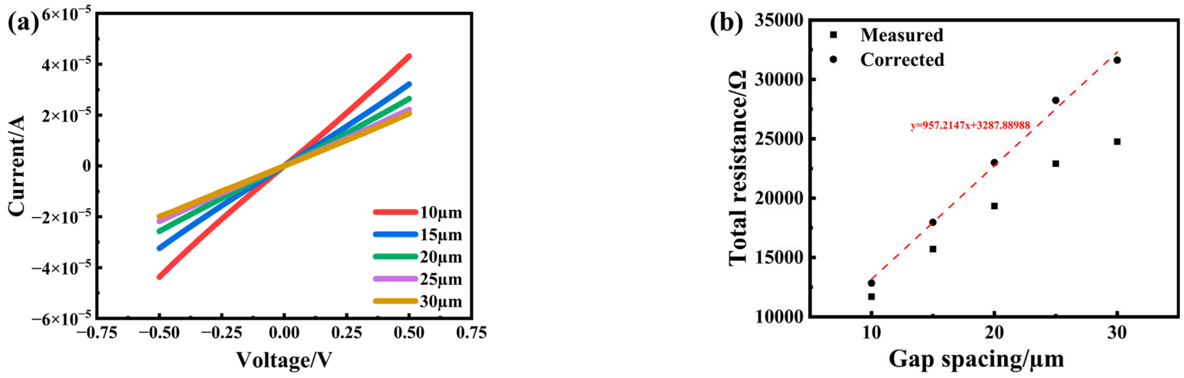

Figure S2. CTLM characteristics of Ti/Pt/Au contacts on B-diamond after vacuum annealing: (a) I–V curves with different gap spacings and (b) extracted total resistance as a function of gap spacing.

The tunneling barrier at the metal/B-diamond contact interface is mainly associated with the potential barrier and depletion region formed at the metal/semiconductor interface. For lightly doped or intrinsic diamond, the carrier concentration near the contact interface is very low, resulting in a wide depletion region and a large injection barrier. This makes hole injection from the metal into diamond difficult and leads to a high contact resistance. In our device, the

boron-doped diamond layer is present at the contact interface. The boron doping increases the hole concentration near the metal/B-diamond interface and narrows the depletion region. The depletion width can be qualitatively expressed as

$$W_D \propto \sqrt{\frac{\varepsilon_s \phi_B}{qN_A}}$$

where W_D is the depletion width, ε_s is the permittivity of diamond, ϕ_B is the effective barrier height, q is the elementary charge, and N_A is the acceptor concentration. Therefore, increasing the boron concentration reduces the tunneling barrier width and enhances hole injection through tunneling or thermionic-field emission. As a result, the contact resistance is reduced and Ohmic-like contact behavior can be obtained.

S2: The subthreshold behavior was evaluated from the $\log_{10}|I_D| - V_{GS}$ transfer characteristics. For the p-channel B-diamond MOSFETs, $|I_D|$ was used, and the subthreshold swing can be estimated by

$$SS = \frac{dV_{GS}}{d(\log_{10}|I_D|)}$$

Based on the selected subthreshold fitting regions, the extracted SS values for MOSFET-I, MOSFET-II, and MOSFET-III are approximately 11.8, 16.4, and 14.0 V/dec, respectively. These relatively large SS values indicate non-ideal subthreshold behavior, which is likely related to the bulk-doped B-diamond channel, PECVD SiO_2/B-diamond interface states, and oxide/interface trap effects. Therefore, in the revised manuscript, the switching behavior is mainly evaluated using the on/off ratio, while the limitation in subthreshold behavior is also acknowledged.

To further evaluate the interface-related properties, forward and reverse C–V measurements were performed from −25 to 25 V, as shown in Figure S3. Due to the voltage limitation of the measurement system and the C–V stretch-out caused by interface-related charges, the device did not fully enter the deep-depletion region. From the measured C–V curve, the maximum oxide capacitance density was extracted as

C_{ox} = 0.0135 μF/cm^2

Considering the actual PECVD SiO_2 thickness of approximately 200 nm, the effective relative dielectric constant of the deposited SiO_2 layer was estimated using

$$\varepsilon_k = \frac{C_{ox} T_{ox}}{\varepsilon_0}$$

where T_{ox} is the oxide thickness and ε_0 is the vacuum permittivity. The calculated ε_r is approximately 3.05, slightly lower than the ideal value of dense SiO_2 (~3.9). This suggests that the PECVD SiO_2 film may have a lower effective film density and that oxide defects or interface-related charges can influence the measured capacitance.

In addition, the C^{-2}–V curve shows a good linear fitting in the depletion-related region. Based on the slope of the C^{-2}–V fitting curve,

$$N_A = \frac{2}{q\varepsilon_0 \varepsilon_r k}$$

where k is the slope of the C^{-2}–V fitting curve, the effective net acceptor concentration of the diamond epitaxial layer was estimated to be 2.09×10^{17} cm^{-3}, which is consistent with the SIMS result. The estimated interface trap charge density is approximately 1.18×10^{12} cm^{-2}.

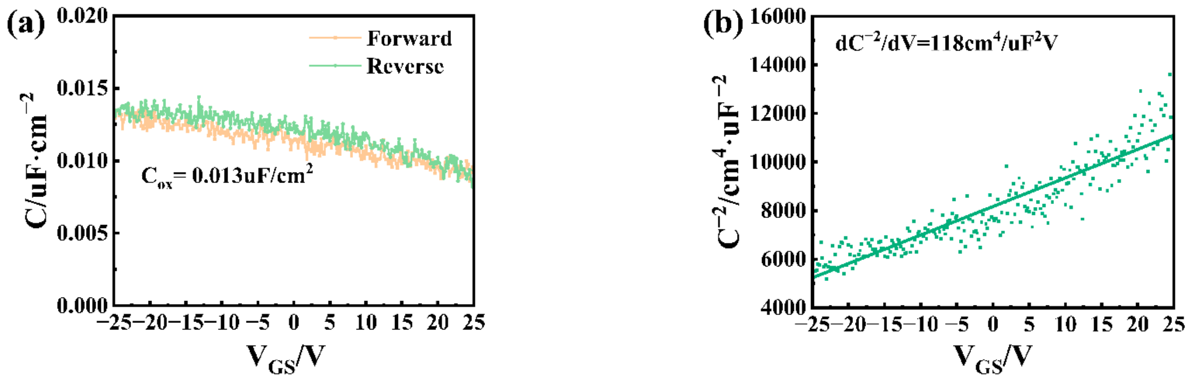

Figure S3 Forward/reverse C–V characteristics and C^{-2}–V fitting of the post-etched B-diamond MOSFET structure.

Furthermore, forward and reverse transfer sweeps were measured to examine the hysteresis behavior, as shown in Figure S4. At the same current level, the reverse-sweep curve is shifted relative to the forward-sweep curve, indicating charge trapping in the PECVD SiO_2 layer or at the SiO_2/B-diamond interface. This result confirms that interface/oxide charge trapping exists in the present device and contributes to the non-ideal transfer characteristics.

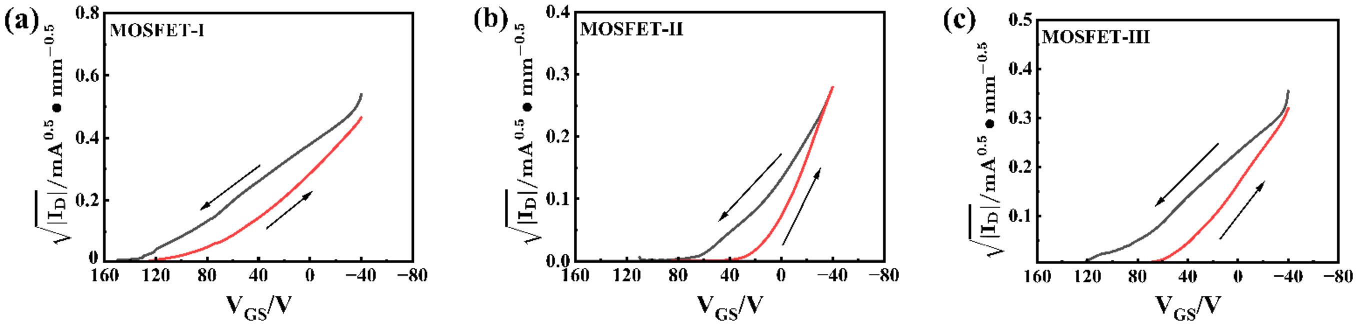

Figure S4 Forward/reverse transfer characteristics of the B-diamond MOSFETs: (a) MOSFET-I; (b) MOSFET-II; and (c) MOSFET-III.

The effective field-effect mobility was also estimated by

$$I_D = \frac{W}{L} C_{OX} \mu [(V_G - V_{th}) V_{DS} - \frac{1}{2} V_D^2]$$

The extracted μ_{FE} values are approximately 0.29, 0.12, and 0.024 cm^2/V·s for MOSFET-I, MOSFET-II, and MOSFET-III, respectively. These values should be regarded as effective or extrinsic field-effect mobility values rather than intrinsic Hall mobility, because they include the effects of access resistance, contact resistance, bulk-doped B-diamond channel conduction, and interface-related trapping at the PECVD SiO_2/B-diamond interface.
